# Effect of Breast and Formula Feeding on Gut Microbiota Shaping in Newborns

**DOI:** 10.3389/fcimb.2012.00094

**Published:** 2012-10-16

**Authors:** Federica Guaraldi, Guglielmo Salvatori

**Affiliations:** ^1^Division of Endocrinology, Diabetology and Metabolism, Department of Internal Medicine, University of TurinTurin, Italy; ^2^Neonatal Intensive Care Unit, Department of Medical and Surgical Neonatology, IRCCS Bambino Gesu’ Children’s HospitalRome, Italy

## Introduction

The gastrointestinal microbiota is a complex and dynamic ecosystem consisting of several hundreds of different microbes, mainly bacteria (10^11–12^ bacteria/g of colonic content, forming 60% of total fecal mass; Eckburg et al., 2005; O’Hara and Shanahan, 2006). Total number of bacteria exceeds 10 times the number of human cells, and the collection of microbial genome (microbiome) contains 100 times more genes than the human genome (Vael and Desager, 2009). Gut microbiota influence the growth and differentiation of gut epithelial cells, and play pivotal nutritive, metabolic, immunological, and protective functions (O’Hara and Shanahan, 2006). Its deregulation is involved in the pathogenesis of immunological, cardiovascular, and metabolic diseases (Hammer, 2011; Maslowski and MacKay, 2011; Harris et al., 2012).

The investigation on microbiota composition started in 1900 (Tissier, 1900) and has been performed by culturing methods since the recent advent of DNA sequence-based methods, that, thanks to their ability to identify a large number of species that cannot be cultivated, have allowed a more complete and rapid assessment of the gastrointestinal ecosystem (Palmer et al., 2007; Adlerberth and Wold, 2009). On the basis of 16S ribosomial – RNA encoding gene, more than 7000 distinct phylotypes have been detected in the human distal gut (Vael and Desager, 2009), with high inter-individual and age variability, but belonging to a limited number of broad taxonomic divisions (mainly the anaerobes *Bacteroides*, *Eubacterium*, *Clostridium*; Hayashi et al., 2002; Eckburg et al., 2005; Zoetendal et al., 2008). In a very recent study, Arumugam et al. (2011), by combining fecal metagenomes of individuals from different countries, identified three different enterotypes (with the prevalence of *Bacteroides*, *Prevotella*, and *Ruminococcus* species, respectively) that are not nation or continent specific, and showed that intestinal microbiota variation is stratified, not continuous, indicating further the existence of a limited number of well-balanced host-microbial symbiotic states. These enterotypes do not seem to differ in functional richness and apparently do not correlate with nationality, gender, age, or body mass index; at the same time, they seem to characterize and be quite stable in individuals, so that they can be restored after perturbations.

Gut microbiota composition and concentration physiologically varies throughout the gastrointestinal tract (increasing gradient from the stomach to the colon and characteristic gut-compartment distribution of microflora) and life stages, progressing from the newborn sterility to the extremely variable and dense colonization of adult gut, under the influence of various internal host-related and external factors (Mackie et al., 1999; Palmer et al., 2007).

## Establishment and Development of Intestinal Microflora

The fetal intestine is sterile and bathed by amniotic fluid. The establishment of the gut microbial population is a continuous and complex process which starts at delivery and proceeds for several years through successive stages under the influence of several internal and external factors (Mackie et al., 1999; Fanaro et al., 2003; Adlerberth and Wold, 2009; Arumugam et al., 2011). Due to the abundance of oxygen in the neonatal gut, facultative aerobes (mainly Enterobacteriaceae, *Enterococcus*, and *Streptococcus* species) represent the first colonizers. *Escherichia coli*, *Enterococcus fecalis*, and *faecium* are the most represented, followed by *Klebsiella* and *Enterobacter*, and, more rarely and transiently, *Aeromonas*, *Pseudomonas*, *Acinetobacter*, alpha-haemolyticus Streptococci, and coagulase-negative Staphylococci (Penders et al., 2006; Adlerberth and Wold, 2009; Vael and Desager, 2009). Their expansion leads to a gradual consumption of oxygen, so to a more reduced environment, which favors the proliferation of obligately anaerobic bacteria, with the dominance of *Bifidobacterium*, *Bacteroides*, and *Clostridium*, followed by *Veillonella*, *Eubacterium*, and *Ruminococcus* species (Penders et al., 2006; Adlerberth and Wold, 2009; Vael and Desager, 2009). With time, anaerobic species will expand and outnumber facultative bacteria (Penders et al., 2006; Adlerberth and Wold, 2009; Vael and Desager, 2009), toward an adult-like microbiota profile, characterized by the preponderance of *Bacteroides* and *Firmicutes*, common occurrence of *Verrucomicrobia* and very low abundance of Proteobacteria and aerobic Gram negative bacteria (Palmer et al., 2007).

Colonizing bacteria derive from the mother (mainly vaginal and intestinal microflora), breast milk (for breast-fed babies), and surrounding environment (which includes equipment, air, other infants, and nursing staff). The pattern and level of exposure during the neonatal period is likely to influence the microbial succession and colonization in the GI tract. Factors influencing microbial colonization can be grouped in two main categories: *extrinsic*, which include geographic area, maternal, and surrounding environment bacteria, mode of delivery, hygiene measures, and feeding habits, and drug therapies; and *intrinsic*, which include the neonatal genetics, bacterial mucosal receptors, and interactions, intestinal pH and secretions, peristalsis, and immune response (Mackie et al., 1999; Penders et al., 2006; Adlerberth and Wold, 2009; Fallani et al., 2010).

Diet has a dominant role over other possible variables such ethnicity, sanitation, hygiene, geography, and climate, in shaping the gut microbiota (De Filippo et al., 2010).

## The Impact of Breast-Feeding on Microbiota Composition

Human milk presents a complex and dynamic composition, influenced by gestational age at parturition, lactation period, and woman’s diet, which differs from formula feeding for nutrients concentrations and composition, and, more importantly, for the exclusive presence of growth factors, cytokines, immunoglobulins, and digestion enzymes (Le Huerou-Luron et al., 2010; Roncada et al., 2012).

Feeding type has been demonstrated to influence microbiota composition directly, by providing the substrates for bacterial proliferation and function (Le Huerou-Luron et al., 2010) and sources of bacterial contamination (originating from the nipple and surrounding skin, and milk ducts for breast milk; from the dried powder, the equipment used for preparation and the water used for suspension for formula milk; Mackie et al., 1999), and indirectly, by modulating the morphology, cell composition and physiology of the intestinal mucosa, and the pancreatic function (Le Huerou-Luron et al., 2010).

Studies performed in the last two decades on large populations of neonates aged ≥4 weeks, using both culturing and molecular methods, demonstrated that Bifidobacteria were the most represented species in both breast- and formula-fed infants (Balmer and Wharton, 1989; Mackie et al., 1999; Harmsen et al., 2000; Fanaro et al., 2003; Bezirtzoglou et al., 2011; Fallani et al., 2011). In most of the cases, no significant count differences were found between breast- and formula-fed infants (Mackie et al., 1999; Harmsen et al., 2000; Fanaro et al., 2003; Fallani et al., 2011). Conversely, Bezirtzoglou et al. (2011) observed more than two times increased numbers of bacteria cells in breast-fed infants, compared to formula-fed ones. Among Bifidobacteria, *Bifidobacterium breve*, *B. adolescentis*, *B. longum*, and *B. bifidum* are isolated in both formula- and breast-fed infants, whereas *B. infantis* is typical of breast-feds, *B. fragilis* of formula-fed infants (Mackie et al., 1999; Penders et al., 2006). In most of the studies, *Bacteroides* and Enterobacteria represent the two most frequently found species after Bifidobacteria (Balmer and Wharton, 1989; Mackie et al., 1999; Harmsen et al., 2000; Fanaro et al., 2003; Fallani et al., 2011). Palmer et al. (2007) and Favier et al. (2002) failed to demonstrate *Bacteroides* as part of the dominant microbiota; this finding could be due to the interfering action of other environmental factors (Penders et al., 2006).

Breast-fed newborns have been demonstrated to carry a more stable and uniform population when compared to the formula-fed ones (Bezirtzoglou et al., 2011). Relatively small amounts of formula supplementation of breast-fed infants will result in shifts from a breast-fed to a formula-fed pattern (Mackie et al., 1999), characterized by a wider microbiota spectrum. In particular, the counts and incidences and counts of *Clostridium* (*C. paraputrificum*, *C. perfringens*, *C. clostridiiforme*, *C. difficile*, and *C. tertium*) and *Streptococcus* (*S. bovis*, *S. faecalis*, and *S. faecium*) species, *Bacillus subtilis*, *Bacteroides vulgatus*, *Veillonella parvula*, *Lactobacillus acidophilus*, *Escherichia coli*, *Pseudomonas aeruginosa* (Benno et al., 1984; Mackie et al., 1999; Penders et al., 2006; Adlerberth and Wold, 2009; Fallani et al., 2010; Bezirtzoglou et al., 2011), *Enterococcus faecalis* (Jimenez et al., 2008; Adlerberth and Wold, 2009), and *Atopobium* (Bezirtzoglou et al., 2011) in the bottle-fed infants were significantly higher than those in the breast-fed infants. On the other hand, *L. rhamnosus* and *Staphylococci* prevailed in breast-fed infants (Adlerberth and Wold, 2009), with *Staphylococcus epidermidis* representing the distinctive tract of the feces of lactating woman and their infant, while it was almost absent in samples from feces of formula-fed infants.

The introduction of solid food profoundly impacts on the microbial ecology of breast-fed infants (Stark and Lee, 1982; Mackie et al., 1999; Adlerberth and Wold, 2009). Once dietary supplementation begins, microbiota profile of breast-fed infants changes toward formula-fed-infants profile, with the significant increase in the count of Enterococci and Enterobacteria, and the appearance of *Bacteroides*, Clostridia, and other anaerobic Streptococci (Stark and Lee, 1982; Mackie et al., 1999; Adlerberth and Wold, 2009). Between the first and the second year of life, differences between breast- and formula-fed infants are lost, and the microbiota profile resembles that of the adult for composition and microbiota counts (Stark and Lee, 1982; Mackie et al., 1999; Adlerberth and Wold, 2009).

## The Impact of Breast-Feeding on Immediate and Long-Term Health-Effects

Numerous studies have been performed in the last decades with the aim to define short- and long-term effects related to the initial microbial gut colonization.

The nature of mucosal microflora acquired in early infancy has been proven to be critical in the determination of mucosal immune response and tolerance, so that alterations of gut environment are directly responsible for mucosal inflammation and disease, autoimmunity, and allergic disorders in childhood and adulthood (Gronlund et al., 2000; Ogra and Welliver, 2008). The type of feeding, through its selective action on bacterial colonization and growth, which, in turn, induce specific T cell responses and modulates substrates oxidation and consumption, has a major impact on the development of immune functions and oral tolerance (Palma et al., 2012). Systematic revisions of available data, pointed out the protective role of breast-feeding against the development of diarrhoea and necrotizing enterocolitis in the newborn (Mackie et al., 1999), and allergic and autoimmune diseases in childhood, including coeliac disease (Akobeng et al., 2006; Palma et al., 2012), type I diabetes and atopic dermatitis, whereas no clear risk reduction was evident in relation with asthma or allergic rhinitis (Bjorksten, 2005; Kramer, 2011). Later in life, breast-feeding has been associated to a reduced risk of inflammatory bowel diseases, cardiovascular diseases, obesity, and type-2 diabetes.

## Potential Protective Role Related to the Addiction of Prebiotics and Probiotics to Formula Food

Because of the recognized healthy properties, exclusive breast-feeding has been recommended by the World Health Organization for the first 6 months of life and supplemental breast-feeding up to 2 years and beyond (Le Huerou-Luron et al., 2010). According to a recent analysis by Le Huerou-Luron et al. (2010), the prevalence of exclusive breast-feeding in the world between 2000 and 2005 was 90% in the early postpartum, but only 41% at 4–6 months of age, with the highest percentages in Africa, followed by East and South Asia, Latin America and The Pacific, and, finally, Europe.

Considerable efforts have been made to mimic the composition of human milk by the addition to formula feeding of living bacteria (*probiotics*), non-digestible fibers, nucleotides and oligosaccharides (*prebiotics*), and bovine lactoferrin in order to induce a breast-fed-similar microbiota colonization in formula-fed infants, with the final aim to stimulate the maturation and proper function of the immune system (Fanaro et al., 2003; Rinne et al., 2005; Singhal et al., 2008; Vael and Desager, 2009). Overall, the implementation of formula food with prebiotics and probiotics has been demonstrated to be effective in changing microflora composition toward the desired breast-feeding pattern and stimulating immune response (Rinne et al., 2005; Sherman et al., 2009). No definitive results are available regarding the real health improvement related to their use (Bjorksten, 2005; Sherman et al., 2009; Vael and Desager, 2009) although in preterm infants their supplementation is associated with a reduced incidence of necrotizing enterocolitis and sepsis (Mackie et al., 1999; Lee, 2011).

## Conclusions

Several studies performed in the past decades have clearly demonstrated the complexity of gut microbiota composition and the modulatory effect played by several endogenous and exogenous factors on it. Type of feeding in the first months of life appears as one of the most important determinants of the child and adult well-being, and its protective action seems to rely mainly on its ability to modulate intestinal microflora composition at early stages of life. In recent years, the implementation of milk formula with prebiotics, probiotics, and lactoferrin has been demonstrated to change newborns’ microflora composition toward breast-feeding pattern and stimulate immune response. At the same time, no definitive results are available regarding the real health improvement, so that breast milk, whose beneficial health-effects are undoubtedly unique, has to be considered the food of choice for infants in the first 6 months of life.

For the same reasons, breast-feeding should be encouraged and, at the same time, new researches are advised in order to better define the composition of intestinal microbial ecosystem and the specific interactions amongst diet, microbiota composition, and children health.
